# Pupil size variations correlate with physical effort perception

**DOI:** 10.3389/fnbeh.2014.00286

**Published:** 2014-08-25

**Authors:** Alexandre Zénon, Mariam Sidibé, Etienne Olivier

**Affiliations:** COSY, Institute of Neuroscience, Université Catholique de LouvainBrussels, Belgium

**Keywords:** pupillometry, effort, psychophysiology, effort-based decision making, pupil dilation, physical exertion

## Abstract

It has long been established that the pupil diameter increases during mental activities in proportion to the difficulty of the task at hand. However, it is still unclear whether this relationship between the pupil size and effort applies also to physical effort. In order to address this issue, we asked healthy volunteers to perform a power grip task, at varied intensity, while evaluating their effort both implicitly and explicitly, and while concurrently monitoring their pupil size. Each trial started with a contraction of imposed intensity, under the control of a continuous visual feedback. Upon completion of the contraction, participants had to choose whether to replicate, without feedback, the first contraction for a variable monetary reward, or whether to skip this step and go directly to the next trial. The rate of acceptance of effort replication and the amount of force exerted during the replication were used as implicit measures of the perception of the effort exerted during the first contraction. In addition, the participants were asked to rate on an analog scale, their explicit perception of the effort for each intensity condition. We found that pupil diameter increased during physical effort and that the magnitude of this response reflected not only the actual intensity of the contraction but also the subjects' perception of the effort. This finding indicates that the pupil size signals the level of effort invested in a task, irrespective of whether it is physical or mental. It also helps refining the potential brain circuits involved since the results of the current study imply a convergence of mental and physical effort information at some level along this pathway.

## Introduction

In addition to its reactivity to light intensity, the diameter of the pupil is affected by many additional parameters which are unrelated to visual stimulation. These include emotions (Partala and Surakka, 2003), attention (Wierda et al., 2012), target detection (Privitera et al., 2010), decision making (Einhäuser et al., 2010), exploration-exploitation trade-off (Jepma and Nieuwenhuis, 2011), and others. However, the most widely recognized cognitive factor influencing pupil size is mental load (Beatty, 1982). Indeed, during mental effort, an increase in pupil diameter has been consistently observed in a very wide variety of tasks (as reviewed in Just et al., 2003), and this increase correlates with the difficulty of the task at hand.

Despite the well-known relation between pupil size and mental effort, surprisingly, the effect of physical effort on pupil size has not been documented so far. Demonstrating such a relationship would help to determine the functional significance of the effort-related pupil response. Here, we attempted to answer three distinct questions: (1) Does the onset of physical effort lead to a systematic pupil response? (2) Does this pupil response vary as a function of the effort intensity? (3) Does this effort-related variation in pupil response signal the perceived effort intensity?

To address the effect of physical effort on pupil size, we asked healthy volunteers to perform a power grip task with different force levels. Since, in addition to the mere effect of the effort intensity on pupil size, we were interested in the relationship between pupil size and the subjective perception of effort, we asked participants to provide subjective ratings of their effort (rate of perceived exertion, or RPE). However, RPE is a subjective measure subject to many potential confounds. So in order to circumvent these limitations, we also gathered other, more implicit measures of effort perception. We offered the possibility to the participants to replicate each effort they performed for a given amount of money. This provided us with two additional measures of effort perception: (1) the acceptance rate, corresponding to the probability with which the participants accepted to replicate the effort, and which is inversely related to the perception of the effort intensity; and (2) the force applied during the effort replication in the absence of feedback that directly depends on how the initial effort, which the participants were attempting to replicate, was perceived. We then evaluated, by means of linear mixed models, how the pupil response measured during the execution of the effort helped to predict these measures of effort perception.

## Methods

### Subjects

A total of 12 young volunteers (seven men and five women, age range 22–31 years) in good physical health were recruited for this study. All participants were right-handed and had a normal or corrected-to-normal vision. None of the subjects had known neurological or psychiatric disorders. Moreover, none of the subjects had suffered in the past from any type of injuries or pain that might have altered their physical performance.

Prior to testing, all subjects provided us with written informed consents. All experimental procedures were approved by the local ethics committees and were in full accordance with the guidelines in the Declaration of Helsinki.

### Procedures

Each subject was tested during three sessions, with a minimum of 2 days between the successive sessions. Participants were also asked to avoid caffeine consumption 2 h before each experimental session. Pupil size was monitored by means of an Eyelink® 1000 plus eyetracker with a 500 Hz sampling rate. The Eyelink was calibrated at the beginning of each session. In addition to the subjects' responses (see below), grip force and pupil size during the task, we recorded flexor digitorum superficialis electromyogram (EMG) activity from surface electrodes.

### Experimental setup

Each subject was comfortably seated in front of a computer screen at a distance of 52 cm. The hip and knee were positioned at 90°. Left forearm and hand were laid, on the table, such that the left flexor digitorum superficialis was relaxed. The right forearm was semi-flexed and in supine position, and the hand was holding a dynamometer. We used a pillow to minimize movements of the forearm, reducing the pain during the force production and assuming a comfortable recuperation between contractions. Subjects had their head resting on a chinrest in order to restrict head movements for pupil size measurements. The room was dimly illuminated and soundproof so that the subjects remained focused on the task. The display and control of the task and the recordings were both performed on the same PC running Matlab (Mathworks®).

### Task

The experience took place in three 4-block sessions repeated on three different days, separated at least by 48 h. Each block consisted of 28 trials and lasted about 4 min. In order to take muscular fatigue into account, we measured the Maximal Voluntary Contraction (MVC) at the beginning of each block, by asking the subjects to squeeze the dynamometer as hard as possible for 3 s. A sound signal marked the beginning and the end of each contraction. This procedure was repeated twice, each followed by the display of a score on the screen, providing the subjects with a feedback about the maximal grip force they exerted. The intensity of all the contractions that the participants had to execute during the subsequent block were defined in proportion to the grip force obtained during these two MVC measurements.

**Figure 1:**
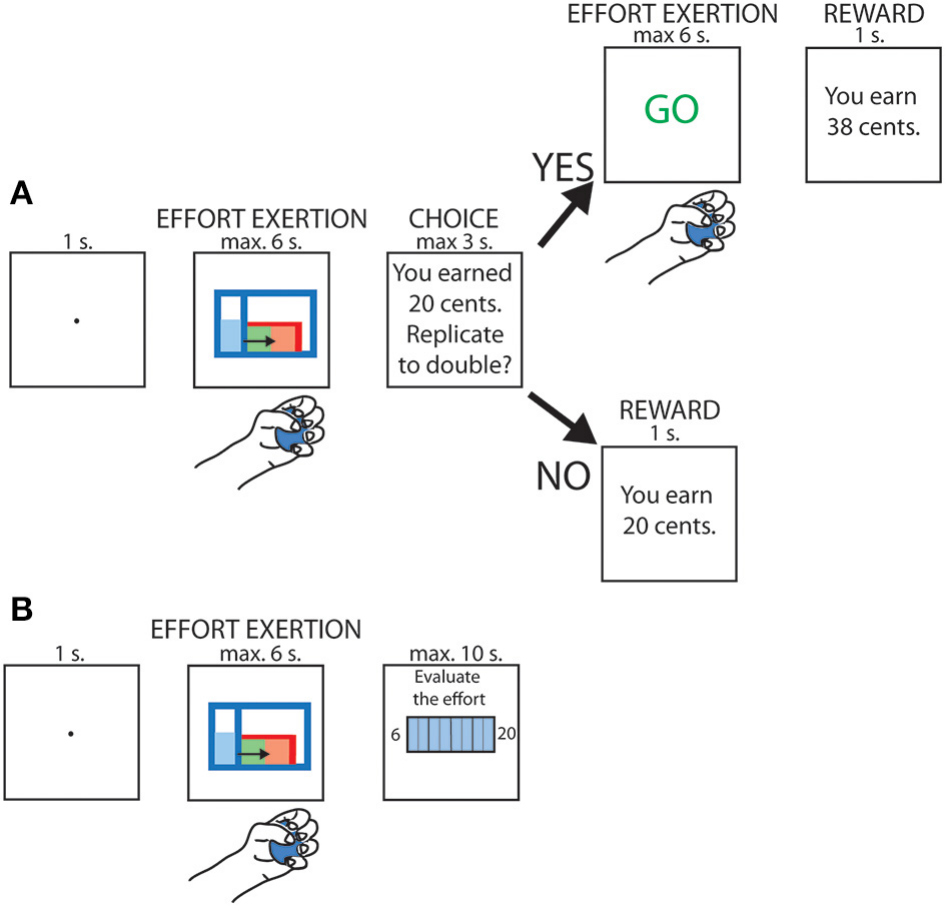
**schematic depiction of the task. (A)** Trials in which replication of the effort was proposed. **(B)** Trials in which subjective rating of the effort was required.

Every subsequent trial was divided into 2 steps (see Figure 1A). First, the subject had to execute an effort by squeezing the dynamometer with a given force; four different levels of force were used and randomized across trials (10, 23, 37 or 50% of the MVC). An online visual feedback about the force currently applied was displayed on the screen, with a gauge level rising along the y axis proportionately to the force exerted by the subject. In order to complete the task, the subjects had to fill a red tank displayed on the screen. As soon as the gauge level exceeded the height of the tank (henceforth referred to as the “threshold”), it started to be filled in green. The task was considered completed when the tank was entirely filled, corresponding to a 3-s long contraction above threshold, whereas the trial was aborted whenever the participant's force remained below threshold for more than 3 s.

A sound signal was used to indicate the beginning and the end of the contraction. All the stimuli displayed on the screen were isoluminant with respect to the background. Isoluminance was ensured by converting the RGB values to luma, according to the coefficients provided in the ITU-R Recommendation BT.709.

After having achieved the first phase of the trial, participants were informed about the reward gained for this first contraction; the reward was varied randomly between 0 and 32 cents. They were then asked whether they wished to replicate the same effort in order to double their reward. Participants responded with the left hand by pressing either the key “Q” on a computer keyboard to decline or “S” to accept. They had maximum 3 s to provide their response. Upon acceptance, they were asked to perform the same contraction a second time but without visual feedback. The final reward depended on the accuracy of the replication. An index of force replication accuracy was computed as follows: 1 − |(Effort1 − Effort2)/(Effort1 + Effort2)|, with Effort1 corresponding to the integral of the grip force during the initial contraction and Effort2 to the integral of the grip force during the effort replication. This measure was then used as a multiplicative factor to determine the final reward.

Finally, four times per block, instead of having to choose whether or not to replicate the previous effort, the participants were asked to rate the perception of the effort they just accomplished by means of a subjective scale (Rate of Perceived Exertion, RPE) shown on the screen (see Figure 1B). This scale ranges from 6 to 20, corresponding to “no effort at all” and “Maximum effort,” respectively (Borg, 1982). The RPE evaluation was performed once per effort condition in each block and the trial during which it occurred was selected pseudo-randomly. Participants had a maximum of 10 s to indicate their perceived level of exertion on the scale.

### Analyses

The pupil size signal was processed to remove the blinks, by applying first the Eyelink® automatic blink detection algorithm and then by checking manually for any missed blink. Blink periods were filled by means of linear interpolation. The signal was band-passed filtered between 0.025 and 25 Hz with a Finite Impulse Response digital filter designed with the window method and down-sampled to 50 Hz. All analyses on pupil size focus on the pupil response to the first contraction of each trial. Only trials in which the participants successfully filled the tank during this first contraction were included in the analyses.

The EMG signal was analyzed by extracting its root-mean square value, with a 200 ms sliding window, and was then log-transformed. To find the onset of the contraction on each trial, we first took the highest positive peak of the derivative of this transformed EMG signal and then selected the first negative derivative value that preceded it. In order to detect the offset of the contraction, because of a lack of complete relaxation following execution of the first contraction, we used the grip force instead of EMG signal. For this analysis, the relative grip force value was used, without taking its derivative, and the offset of the contraction was taken as the time following completion of the contraction, when relative grip force fell below 5% of the MVC value.

Statistical analyses were performed with Matlab® for the one-way ANOVAs and the R software for the generalized linear mixed models. For these latter analyses, the subject indexes and the order of the sessions were included as random factors in the models. We first compared different random models, increasing gradually the number of random parameters (i.e., random slopes). When adding more parameters failed to improve the model significantly (according to a chi-square test on deviance Bolker et al., 2009), we stopped the procedure. All the possible fixed effects in the model (including all possible combinations of factors and their interactions) were then compared by means of an automated method based on the Akaike Information Criterion (library glmulti, Calcagno and de Mazancourt, 2010). This allowed us to keep in the model only the factors that were relevant to account for the data, permitting to maximize the power of our analyses. We ensured that the correlations between fixed effects remained under 0.6 to avoid multicollinearity issues, which would have impeded our ability to interpret the slope coefficients.

## Results

### Effect of the onset and offset of the first contraction on pupil size

**Figure 2:**
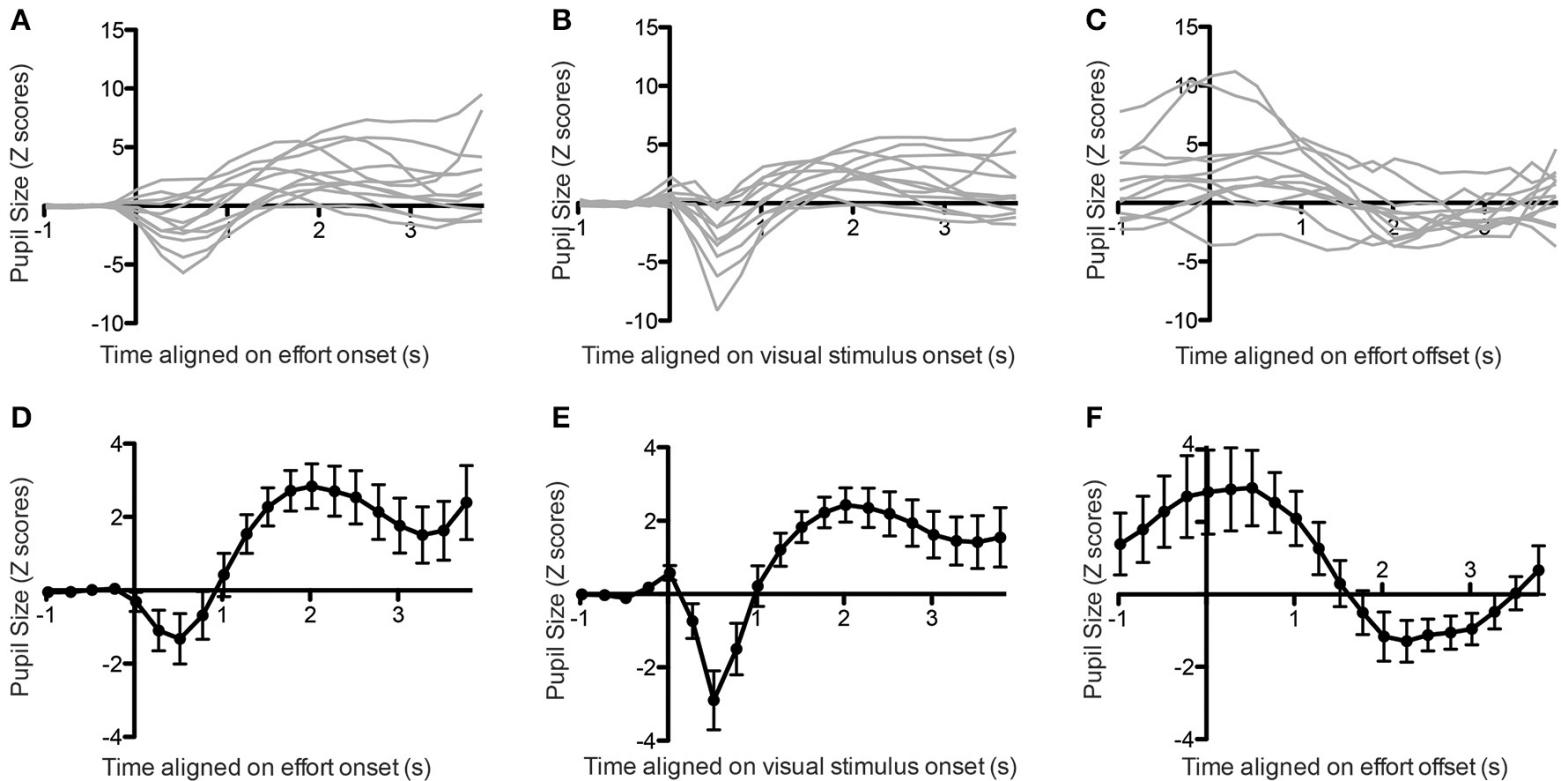
**Pupil size aligned on effort onset (A, individual subjects, D, average), stimulus onset (B, individual subjects, E, average) or effort offset (C, individual subjects, F, average)**. Error bars indicate standard error of the mean.

We first analyzed the pupil size aligned on the onset of the first contraction. For each trial, we measured the pupil size starting 1 s before the contraction onset up to its completion, with a maximum duration of 4 s. The pupil size signals were then transformed to Z scores by subtracting the mean and dividing by the standard deviation of the signal acquired during the 1-s baseline period prior to contraction onset, and were binned in twenty 250 ms bins, for a total duration of 5 s. In all but one subject, we found a significant increase in pupil size following the contraction onset (one-way ANOVA on 20 time bins, all *p*-values for main effect of time < 0.0001). In six subjects, the increase in pupil size was preceded by an initial dip (Tukey *post-hoc* tests, *p* < 0.05; see Figures 2A,D).

Previous studies have linked a similar initial dip in pupil size to the visual stimulation (Bradley et al., 2008; Privitera et al., 2010). In order to confirm that this was also the case in the present study, we looked at the pupil response aligned on the onset of the visual stimulation, instead of the contraction onset. In this case we found a much clearer initial dip for all subjects, confirming that it originated from the visual stimulation (all *p* < 0.05; see Figures 2B,E).

We then applied the same method to look at pupil changes following contraction offset. We took the pupil size signal from 1 s before offset up to the initiation of the next contraction, with a maximum of 4 s. In all subjects we found that pupil size decreased following the offset of contraction, and this effect was significant in 10 out of the 12 subjects (Figures 2C,F, main effect of time on pupil size: all *p* < 0.0001).

### Dependency on effort condition

**Figure 3:**
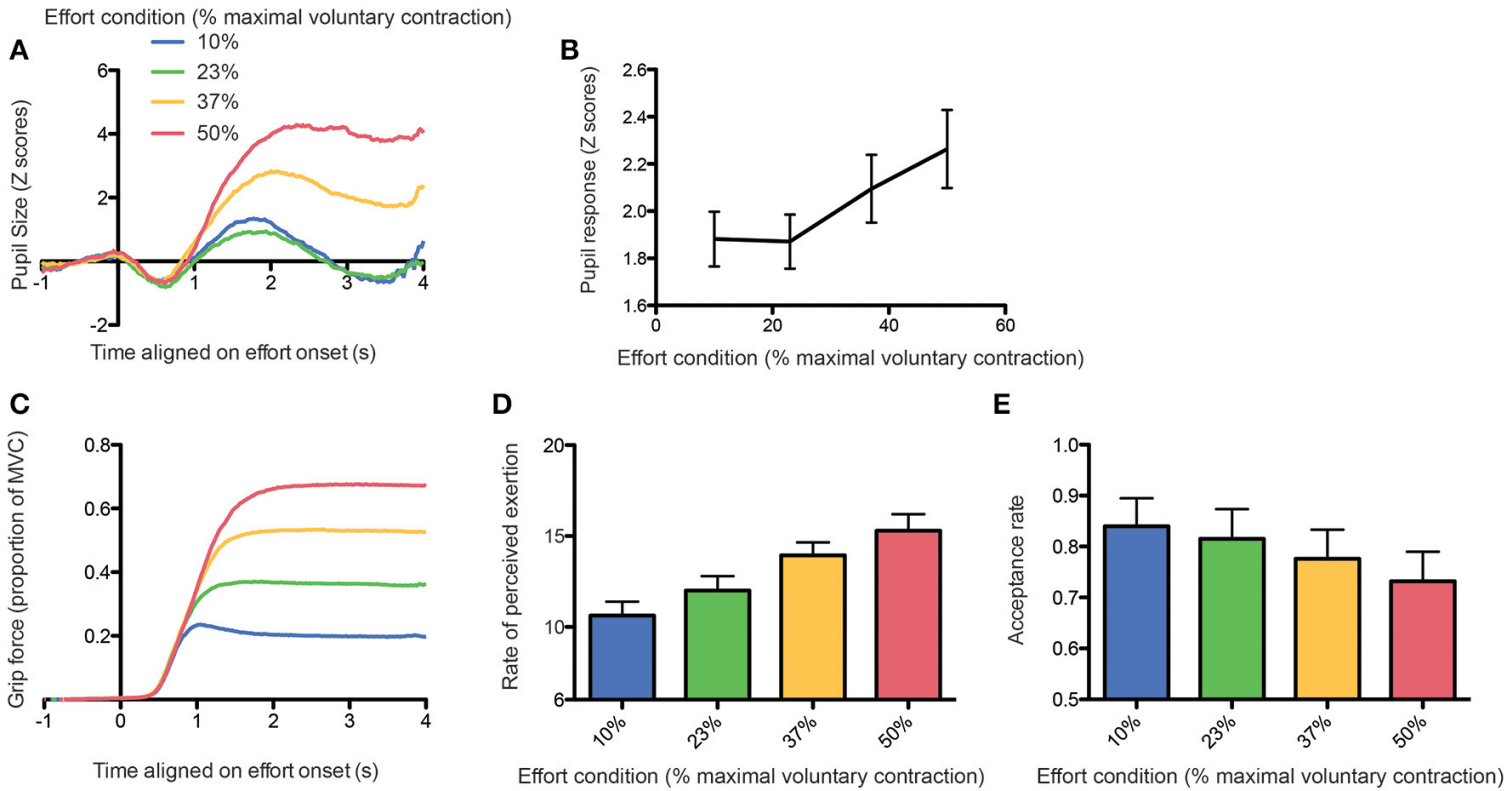
**(A)** Average pupil size response aligned on effort onset as a function of the effort condition. **(B)** Peak-to-peak amplitude of the pupil response as a function of the effort condition. **(C)** Grip force aligned on effort onset as a function of the effort condition. **(D)** Rate of perceived exertion (RPE) as a function of the effort condition. **(E)** Acceptance rate as a function of the effort condition.

Figure 3A shows the pupil response aligned with the onset of the first contraction as a function of the effort condition. There was clearly a larger increase in pupil size with larger efforts, except for the effort condition corresponding to 23% of the MVC, which did not differ from the 10% effort condition. In order to quantify this finding, we ran a one-way repeated-measure ANOVA with the log-transformed peak-to-peak pupil response amplitude (defined as the difference between the minimal and maximal pupil size during the 4 s following contraction onset) as dependent variable and the effort condition as independent variable (see Figure 3B). The main effect of effort condition was significant [*F*_(3, 11)_ = 86.6, *p* < 0.0001] and Tukey *post-hoc* tests confirmed that 10 and 23% effort conditions did not differ from each other.

This lack of difference between these two conditions could be explained by a poor compliance of the participants to follow the instructions, whom could exert more force than required in the 10% MVC condition, thereby suppressing the difference between the 2 lower effort conditions. In order to exclude this possibility, we looked at the grip force actually applied during task execution. We found that subjects followed instructions correctly and that the grip force was proportional to the level of force required, with an additional safety margin (see Figure 3C, RM-ANOVA: main effect of effort condition on average grip force: [*F*_(3, 11)_ = 5787.25, *p* < 0.0001; all Tukey tests were significant with *p*-values < 0.0001].

We also confirmed that the subjective perception of effort by participants was proportional to effort conditions [one-way RM-ANOVA on RPE: *F*_(3, 11)_ = 197.29, *p* < 0.0001, see Figure 3D), with a significant difference already present between the 10 and 23% conditions (Tukey *post-hoc* test, *p* < 0.0001). Finally, we found that the probability to accept the effort replication decreased significantly with increasing effort requirements, including between the 10 and 23% effort conditions (generalized linear mixed model, all *p*-values < 0.01; see Figure 3E).

### Correlations with effort parameters

**Figure 4:**
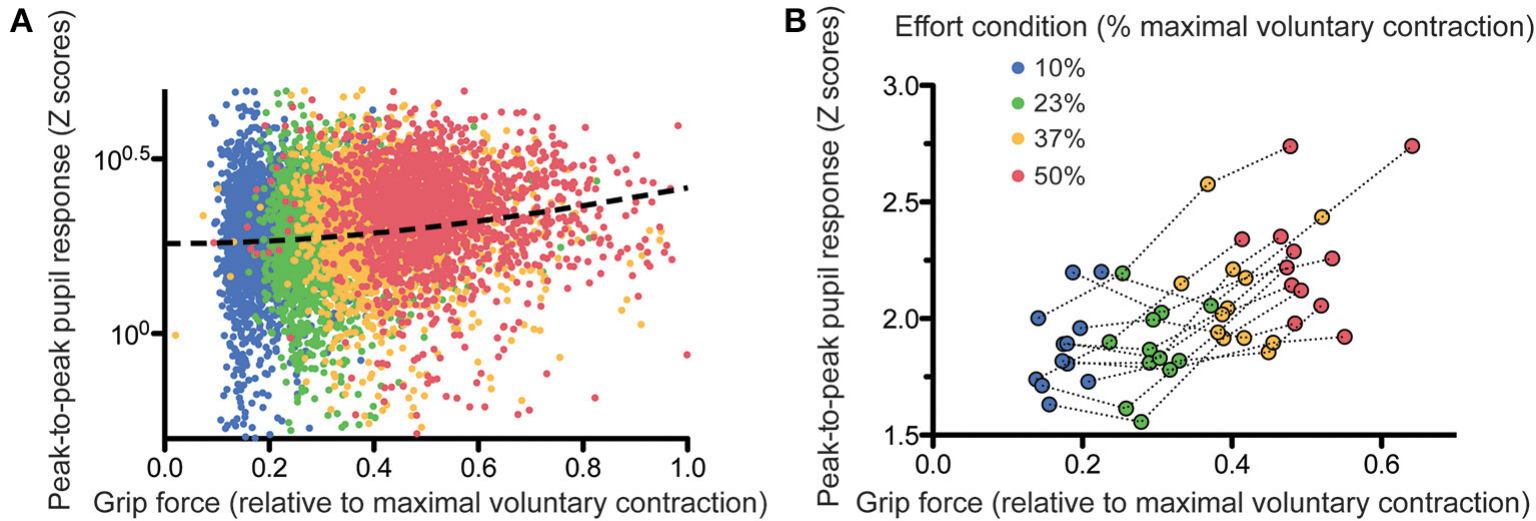
**Relation between grip force and pupil size response, trial-by-trial (A) and subject-by-subject (B)**. The dashed line on **(A)** shows the quadratic fit on the data.

We then looked directly at the correlation between pupil response amplitude and effort parameters. In order to take account of the non-linearity of the grip-force—pupil response relationship, we ran quadratic regressions between peak-to-peak pupil response amplitude (log-transformed) and grip force. When performed subject by subject, these analyses provided significantly positive slopes in 8 out of the 12 subjects (*p* < 0.05). When performed on the whole population of subjects, the regression was highly significant (see Figure 4A including all trials or Figure 4B showing the average pupil response for each subject in each effort condition; slope coefficient = 0.804 ± 0.199, *t* = 4.025, *p* < 0.0001).

In order to determine to what extent the pupil response reflected the perception of effort, we examined how effort replication could be predicted by the pupil response. Obviously, since the perception of effort (i.e., how effortful the participants perceive the contraction to be) is proportional to the intensity of the contraction (i.e., the actual force exerted during the contraction) and that pupil size also depends on the contraction intensity, we can infer that pupil size will be strongly correlated with the perception of effort. However, we wanted to determine whether pupil size is a marker of the intensity of a physical effort, or of how effortful the subject perceives it to be. If pupil size depends on effort perception and not only on physical effort intensity, then pupil size should provide some information, in addition to the actual grip force applied during the first contraction, allowing us to predict the level of grip force during the effort replication. This is because the effort applied during the replication depends on how effortful the participants perceived the first contraction to be. We devised a linear mixed model including first grip force and a second degree orthogonal polynomial of the pupil response (to account for the non-linear relationship shown above) during the first contraction as predictors and grip force during effort replication as dependent variable. Automatic model selection (see Methods and Supplementary results) found that the best statistical model was the one including the main effects of the first contraction and the pupil response, with their interaction. In addition to the trivial correlation between first and second grip force levels, a significant positive correlation between the linear term of the pupil response and the second grip force was observed (slope = 1.73 ± 0.45, *t* = 3.80, *p* < 0.0001). This indicates that, irrespective of the intensity of the first contraction, variations of pupil size correlated with the amount of force applied during the effort replication, and hence, with effort perception. We also found a significant interaction between the quadratic term of the pupil size and the first grip force (slope = −1.18 ± 0.50, *t* = −2.329, *p* = 0.01). This effect is more difficult to interpret but seems to indicate that the relation between pupil size and effort perception might vary as a function of force intensity.

We applied the same procedure with the RPE, which represents a more explicit means of estimating effort perception. In this case, the best linear mixed model included again the main effect of pupil response during the first contraction, the grip force and their interaction (see Supplementary Results). There was a significant correlation between pupil response (especially its quadratic component) during the first effort and its subjective estimation as measured with the RPE (slope = 1.93 ± 0.80, *t* = 2.42, *p* = 0.007), confirming that pupil size could be regarded as a marker of subjective effort perception.

Finally, following a similar reasoning, we tried to determine whether pupil response, after factoring out the effect of the physical effort exerted, was predictive of the decision of the subject to replicate the effort or not. Again we applied the same analysis, now including effort replication acceptance as a binary dependent variable, and effort intensity, the reward proposed for replication and the pupil response during the first contraction as predictors. The best model included all these main effects, and the interaction between reward and pupil size (see Supplementary Results). In addition to the expected effects of the first grip force (*p* < 0.0001) and of reward (*p* < 0.0001), we also found a significant negative slope between pupil size increase during the first contraction and the probability of accepting to replicate this effort (slope = −17.85 ± 6.70, *Z* = −2.664, *p* = 0.007).

## Discussion

In the present study we found that pupil size increases systematically during physical effort. Most interestingly, our results show that pupil size is a good indicator of the actual intensity of the physical exercise and of how effortful it is perceived to be.

### Properties of the pupil response

Participants showed an initial decrease in pupil size at the onset of the visual stimulus. This dip has also been documented in previous studies on mental effort (Beatty, 1982), sometimes associated with an initial light reflex caused by image display onset (Bradley et al., 2008). In the present case, the fact that the display was isoluminant suggests that other aspects than the brightness of the stimulus caused the pupil response, such as a change in spatial frequency, color saturation or contrast (Privitera et al., 2010). However, exact isoluminance is very difficult to achieve and we cannot exclude that slight changes in the luminance of the stimuli could have had some impact on this initial pupil response.

During the execution of the effort, the magnitude of the changes in pupil diameter did not follow grip force linearly. Indeed, the pupil responses obtained during the two lower effort conditions were indistinguishable. This was not due to a difference in how effortful these conditions were perceived to be, as shown by the linear relation observed between the RPE and the probability to replicate the effort. Instead, it suggests a non-linear convex relation between effort and pupil size. Interestingly, studies on mental load have also reported similar non-linear relations between pupil response and parameters linked to task difficulty, such as the number of digits to store in memory (Beatty, 1982).

### Pupil size and the evaluation of effort

The relationship existing between pupil size and mental effort has been known for decades (Hess and Polt, 1964; Beatty, 1982). However, this is merely a correlative observation and the factors that cause this pupil response remain undetermined. In most studies having measured pupil size during cognitive tasks, mental effort was manipulated by increasing task difficulty. Increases in task difficulty led to increases in pupil size but it is unclear whether these changes are related to the intensity of the mental activity itself (e.g., increase in working memory load) or if it is signaling how effortful it is perceived by the subject. Our finding that pupil size varies also commensurately to physical effort perception argues in favor of the latter hypothesis.

In order to determine whether, in addition to a correlation with the actual physical effort intensity, pupil size correlated with the effort perception, we performed linear mixed models with both grip force and variables indicative of the evaluation of the effort. Any significant effect of the effort perception variables found in this context means that pupil response correlates with the perception of effort, over and above the actual physical intensity of the effort.

We must acknowledge that grip force contractions during the present study could have been associated with variations of mental activity. If these changes in mental effort were proportional to the force exerted, then it could be argued that the pupil responses we observed were in fact related to the concurrent mental activity instead of the physical effort itself. Nevertheless, we consider this hypothesis very unlikely. Indeed, the only demanding cognitive task occurred at the time of the choice of whether or not to accept the replication of the effort. This choice followed the pupil response and could not have possibly influenced it. During the contraction itself, the subjects had only to monitor the gauge on the screen, in order to maintain the level of the contraction. This very low-demand visual task was identical for all the effort levels, and could not consequently account for the relation between pupil responses and effort intensity.

### Neurophysiological mechanisms

Mental and physical efforts correspond to two very distinct phenomena; the former is associated with the allocation of cognitive resources while the latter is associated with muscle contraction. Yet, they share many common features and lead to similar bodily responses.

Prolonged mental and physical efforts both induce fatigue (DeLuca, 2005; Marcora et al., 2008), and mental fatigue leads to decrements in physical performance (Marcora et al., 2009). Mental and physical fatigue often co-exist in clinical disorders, such as Parkinson Disease, multiple sclerosis or chronic fatigue syndrome (reviewed in DeLuca, 2005). Mental and physical exertion lead also both to similar catecholamine (Fibiger and Singer, 1984; Fibiger et al., 1984) and cardiovascular responses (Backs and Seljos, 1994; Williamson et al., 2006). The current findings add to this list the observation that both mental and physical efforts lead to proportional increases in pupil size.

This relationship between pupil response and these two seemingly different behavioral factors raises the question of the mechanisms involved. A common denominator to these two phenomena could be the activation of the autonomic nervous system, which is known to occur during both physical and mental activity (Blatt, 1961; Goldstein and Shapiro, 1988; Seals, 1993; Kluess et al., 2000). Indeed, the ciliary ganglion, responsible for pupil constriction, is part of the parasympathetic autonomic nervous system, and receives its inputs from the parasympathetic preganglionic Edinger-Westphal nucleus, while pupil dilation is under the control of the sympathetic Superior Cervical Ganglion (Szabadi, 2013). Even though the existence of a unitary autonomic response, or orienting reflex, has been falsified by experimental evidence (Barry, 2006), and the responses of its different effectors often do not correlate with one another (Taylor and Epstein, 1967), it is possible that the autonomic response caused specifically by the execution of effort includes pupil dilation as one of its effectors. The link between autonomic activation and pupil dilation could be the Locus Coeruleus (LC), a structure at the origin of most noradrenergic projections in the human brain and known to be part of the central autonomic nervous system (Szabadi, 2013). Spontaneous and drug-induced changes in arousal are accompanied by both modifications of the Locus Coeruleus activity and baseline pupil diameter variations (discussed in Gilzenrat et al., 2010). In addition, a correlation between pupil size and Locus Coeruleus BOLD activity has been found in functional imagery (Sterpenich et al., 2006) and pupil size correlates with the discharge rate of the neurons in the LC (Aston-Jones and Cohen, 2005). In terms of the circuits involved, LC provides direct inhibitory inputs to the Edinger-Westphal nucleus in cats (Breen et al., 1983), but seemingly not in primates (Steiger and Büttner-Ennever, 1979). So rather than an inhibition of pupil constriction, LC activation could lead to excitation of the sympathetic circuit responsible for pupil dilation, through its α1-adrenergic connections with the spinal preganglionic nuclei (Szabadi, 2013). Future research that would monitor the other autonomic variables that also depend on LC activity during the performance of physical efforts of different intensities could help refining our understanding of the link between effort, pupil dilation, the LC and the autonomic nervous system.

In addition to the LC, pupil size is also known to be affected by cortical inputs (Wilhelm et al., 2002), likely to be responsible for some of the pupil responses to high-level cognitive features (Qiyuan et al., 1985; Wierda et al., 2012; Naber and Nakayama, 2013; Cavanagh et al., 2014). Potential cortical candidates could be the structures that show activities varying according to both mental and physical effort intensity. A functional imagery study showed that ventral striatum is active during both a Stroop and a grip force task and that this activity followed the degree of engagement in either task (Schmidt et al., 2012). Similarly, the activity of the amygdala (Gur et al., 1997; Floresco and Ghods-Sharifi, 2006; Schaefer et al., 2006) and Anterior Cingulate Cortex (Walton et al., 2003; Mulert et al., 2005; Floresco and Ghods-Sharifi, 2006; Esposito et al., 2009), which both project to ventral striatum, has also been linked to physical and mental effort. These findings suggest that the limbic-striatal circuit could also potentially be at the origin of the effort-related pupil signal.

In conclusion, the present findings show that pupil size tracks the level of effort invested in a task, irrespective of whether it is mental or physical. This implies that mental and physical effort signals converge, at some level, to the circuit responsible for pupil dilation, which helps us to refine the potential structures belonging to this circuit. This also confirms that pupil size measurement can be used as a valid marker of effort in a wide variety of tasks.

### Conflict of interest statement

The authors declare that the research was conducted in the absence of any commercial or financial relationships that could be construed as a potential conflict of interest.
