# Supplementary material for: Pupil size variations correlate with physical effort perception
Source: Front Behav Neurosci. 2014 Aug 25;8:286. doi: 10.3389/fnbeh.2014.00286 (PMC4142600; doi:10.3389/fnbeh.2014.00286)
Supplement: Supplementary file 1 [file DataSheet1.PDF]

Supplementary results

Predictive effect of pupil size on force replication

Force replication: gf2
Force during first contraction: gf1
Orthogonal second degree Polynomial of pupil size: pu
    Linear term: pu1
    Quadratic term: pu2
Subject index: suj
Session number: sessions

Random effects are selected manually before running glmulti, by gradually adding parameters until the improvement in deviance is no longer significant, according to a chi square test.

Best model:

Linear mixed model fit by maximum likelihood
Formula: gf2 ~ 1 + gf1 + pu + pu:gf1
Data: data

AIC	BIC	logLik	deviance	REMLdev
5821	5905	-2897	5795	5802

Random effects:

Groups	Name	Variance	Std.Dev.	Corr
suj	(Intercept)	0.09009325	0.300155	
	gf1	0.00782822	0.088477	0.594
sessions	(Intercept)	0.00190016	0.043591	
	gf1	0.00092512	0.030416	-0.233
Residual		0.18767782	0.433218	

Number of obs: 4887, groups: suj, 12; sessions, 3

Fixed effects:

	Estimate	Std. Error	t value	p value
(Intercept)	-0.007542	0.090462	-0.083	4.7e-01
gf1	0.792212	0.031730	24.967	4.0e-122 ***
pu1	1.727249	0.454321	3.802	7.1e-05 ***
pu2	0.362119	0.451071	0.803	2.1e-01
gf1:pu1	0.214083	0.462001	0.463	3.2e-01
gf1:pu2	-1.178948	0.506160	-2.329	1.0e-02 *

Signif. codes: ***: 0.0 - 0.001; **: 0.001 - 0.01; *: 0.01 - 0.05; . : 0.05 - 0.1

Correlation of Fixed Effects:

	(Intr)	gf1	pu1	pu2	gf1:p1
gf1	0.422				
p1	-0.001	-0.025			

p2	0.000	0.011	-0.033		
gf1:p1	-0.006	0.001	0.017	0.044	
gf1:p2	0.002	0.007	0.020	0.199	0.107

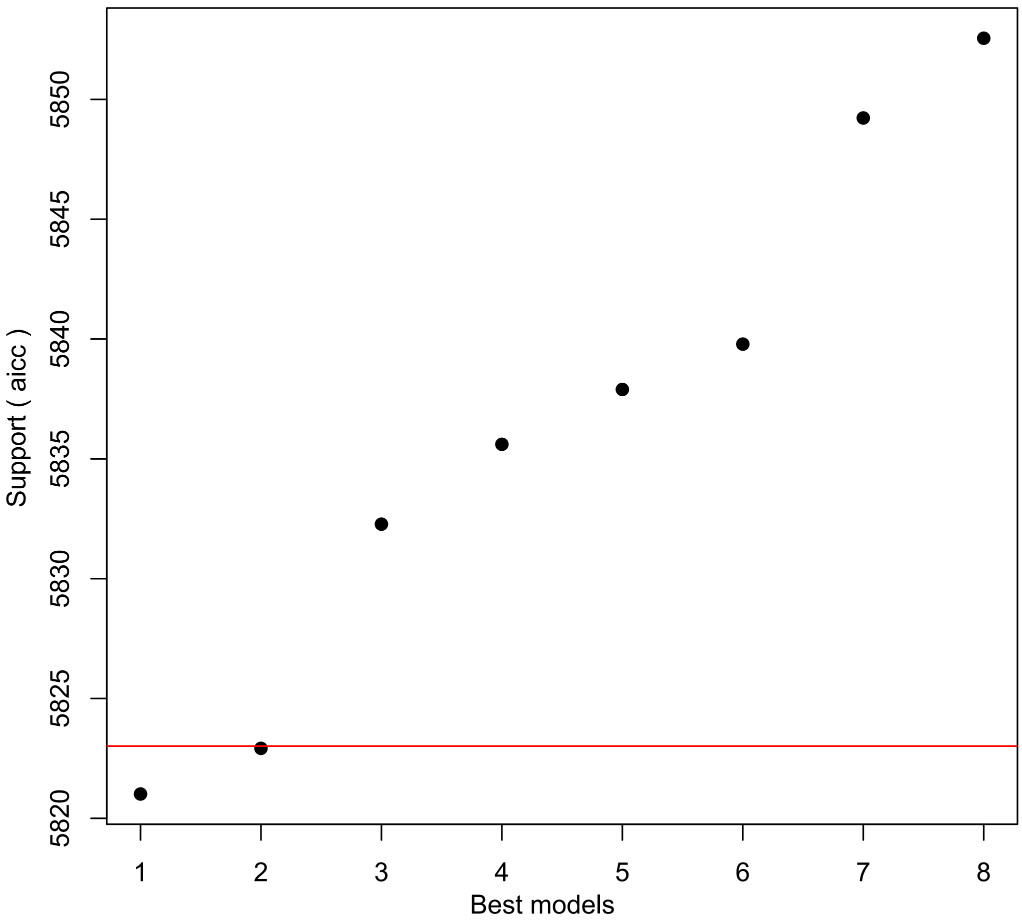

[[1]] gf2 ~ 1 + gf1 + pu + pu:gf1
[[2]] gf2 ~ 1 + gf1 + pu
[[3]] gf2 ~ 1 + gf1 + pu:gf1
[[4]] gf2 ~ 1 + gf1
[[5]] gf2 ~ 1 + pu + pu:gf1
[[6]] gf2 ~ 1 + pu
[[7]] gf2 ~ 1 + pu:gf1
[[8]] gf2 ~ 1

Predictive effect of pupil size on RPE

RPE: rpe
Force during first contraction: gf1
Orthogonal second degree Polynomial of pupil size: pu
    Linear term: pu1
    Quadratic term: pu2
Subject index: suj
Session number: sessions

Random effects are selected manually before running glmulti, by gradually adding parameters until the improvement in deviance is no longer significant, according to a chi square test.

Best model:

Linear mixed model fit by maximum likelihood
Formula: rpe ~ 1 + gf1 + pu + pu:gf1

AIC	BIC	logLik	deviance	REMLdev
2691	2746	-1334	2669	2669

Random effects:

Groups	Name	Variance	Std.Dev.	Corr
suj	(Intercept)	0.092397	0.303969	
	gf1	0.047431	0.217787	0.213
sessions	(Intercept)	0.005424	0.073648	
Residual		0.605318	0.778022	

Number of obs: 1117, groups: suj, 12; sessions, 3

Fixed effects:

	Estimate	Std. Error	t value	p value
(Intercept)	0.004158	0.100486	0.041	0.48350
gf1	0.540884	0.067828	7.974	7.7e-16 ***
pu1	1.105151	0.833555	1.326	0.09249 .
pu2	1.936544	0.797984	2.427	0.00762 **
gf1:pu1	1.229568	0.849970	1.447	0.07400 .
gf1:pu2	-1.275963	0.792258	-1.611	0.05364 .

Signif. codes: ***: 0.0 - 0.001; **: 0.001 - 0.01; *: 0.01 - 0.05; . : 0.05 - 0.1

Correlation of Fixed Effects:

	(Intr)	gf1	pu1	pu2	gf1:pu1
gf1	0.178				
pu1	-0.004	-0.052			
pu2	0.004	0.023	-0.037		
gf1:pu1	-0.025	-0.009	0.038	-0.057	
gf1:pu2	0.005	0.002	-0.057	0.064	0.090

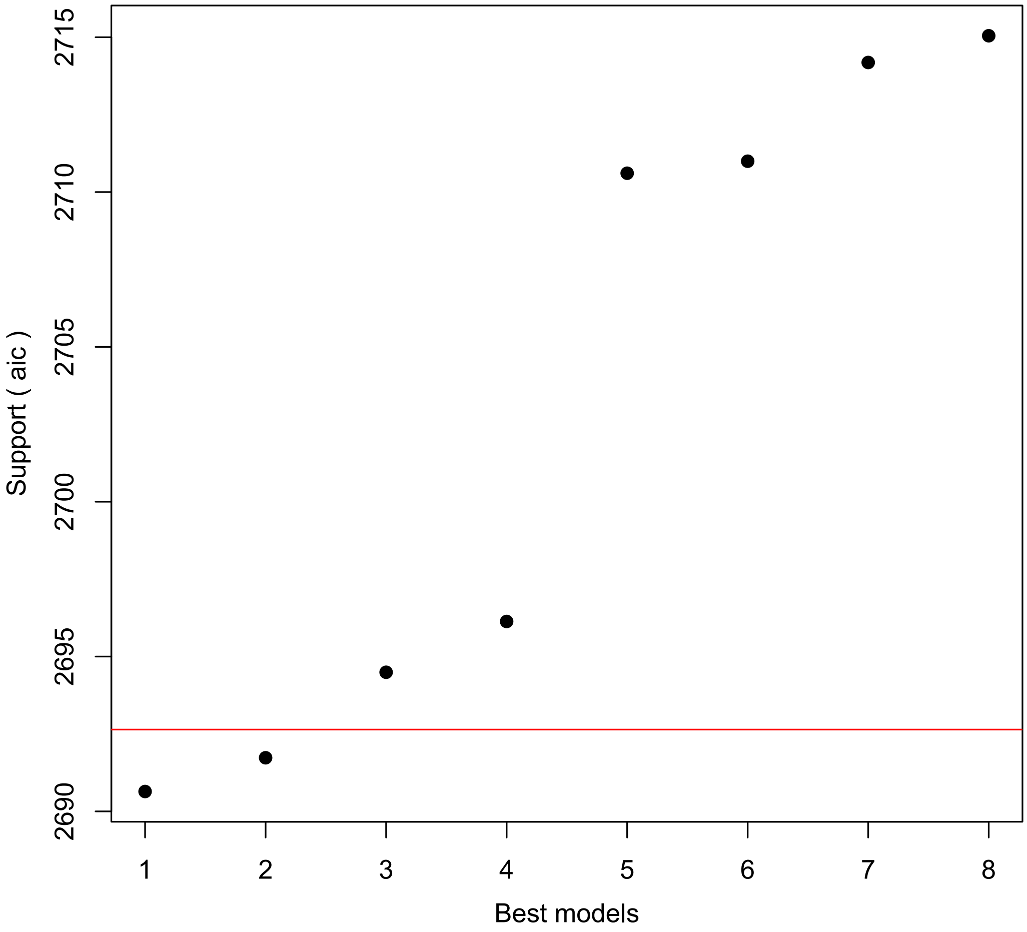

[[1]] rpe ~ 1 + gf1 + pu + pu:gf1
[[2]] rpe ~ 1 + gf1 + pu
[[3]] rpe ~ 1 + gf1 + pu:gf1
[[4]] rpe ~ 1 + gf1
[[5]] rpe ~ 1 + pu + pu:gf1
[[6]] rpe ~ 1 + pu
[[7]] rpe ~ 1 + pu:gf1
[[8]] rpe ~ 1

Effect of pupil size on acceptance rate

Acceptance rate: accepted
Force during first contraction: gf1
Reward proposer: r
Orthogonal second degree Polynomial of pupil size: pu
Subject index: suj
Session number: sessions

Random effects are selected manually before running glmulti, by gradually adding parameters until the improvement in deviance is no longer significant, according to a chi square test.

Best model:

Generalized linear mixed model fit by the Laplace approximation
Formula: accepted ~ 1 + r + pu + gf1 + pu:r

AIC	BIC	logLik	deviance
3069	3208	-1514	3029

Random effects:

Groups	Name	Variance	Std.Dev.	Corr
suj	(Intercept)	7.01415	2.64842	
	gf1	0.55751	0.74666	-0.391
	r	6.56840	2.56289	0.791 -0.469
	gf1:r	0.49108	0.70077	-0.304 0.976 -0.499
sessions	(Intercept)	1.34185	1.15838	
	r	0.54677	0.73944	0.988

Number of obs: 7825, groups: suj, 12; sessions, 3

Fixed effects:

	Estimate	Std. Error	z value	Pr(>|z|)
(Intercept)	5.02130	1.01173	4.963	6.94e-07 ***
r	5.01939	0.80993	6.197	5.74e-10 ***
p1	-17.85027	6.69997	-2.664	0.00772 **
p2	2.53042	6.46601	0.391	0.69554
gf1	-0.39461	0.07611	-5.185	2.16e-07 ***
r:p1	-9.46369	7.15215	-1.323	0.18577
r:p2	13.16030	7.58149	1.736	0.08259 .

Signif. codes: ***: 0.0 - 0.001; **: 0.001 - 0.01; *: 0.01 - 0.05; . : 0.05 - 0.1

Correlation of Fixed Effects:

	(Intr)	r	p1	p2	gf1	r:p1
r	0.845					
p1	-0.017	-0.026				
p2	0.004	0.006	-0.082			
gf1	-0.233	0.002	-0.008	0.004		
r:p1	-0.018	-0.028	0.736	-0.078	0.049	
r:p2	0.003	0.008	-0.079	0.674	0.013	0.016

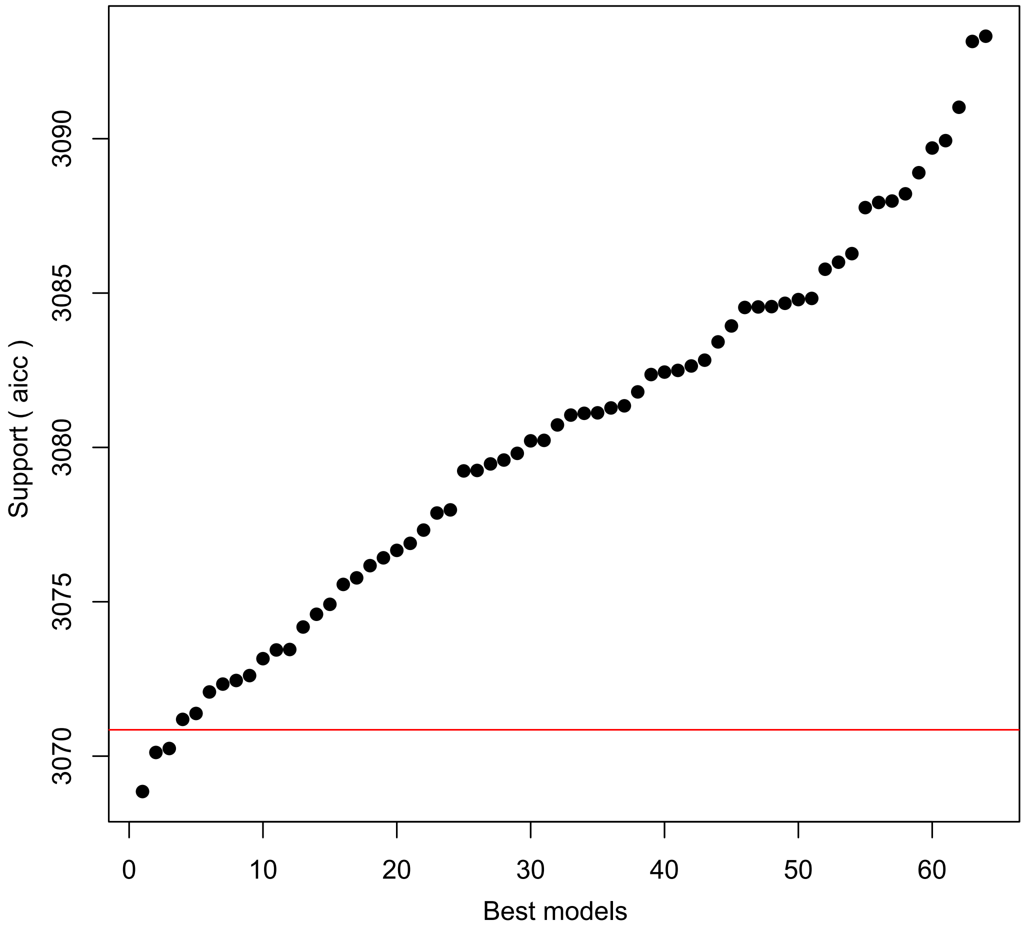

[[1]] accepted ~ 1 + r + pu + gf1 + pu:r
[[2]] accepted ~ 1 + r + pu + gf1 + pu:r + gf1:r
[[3]] accepted ~ 1 + r + pu + gf1
[[4]] accepted ~ 1 + r + pu + gf1 + pu:r + gf1:pu
[[5]] accepted ~ 1 + r + pu + gf1 + gf1:r
[[6]] accepted ~ 1 + r + gf1 + pu:r
[[7]] accepted ~ 1 + r + gf1
[[8]] accepted ~ 1 + r + pu + gf1 + pu:r + gf1:r + gf1:pu
[[9]] accepted ~ 1 + r + pu + pu:r + gf1:r
[[10]] accepted ~ 1 + r + gf1 + pu:r + gf1:r
[[11]] accepted ~ 1 + r + gf1 + gf1:r
[[12]] accepted ~ 1 + r + pu + gf1 + gf1:pu
[[13]] accepted ~ 1 + r + pu + gf1:r
[[14]] accepted ~ 1 + r + pu + gf1 + gf1:r + gf1:pu
[[15]] accepted ~ 1 + r + pu + pu:r + gf1:r + gf1:pu
[[16]] accepted ~ 1 + r + gf1 + pu:r + gf1:pu
[[17]] accepted ~ 1 + r + gf1 + gf1:pu
[[18]] accepted ~ 1 + r + pu:r + gf1:r
[[19]] accepted ~ 1 + r + gf1:r
[[20]] accepted ~ 1 + r + gf1 + pu:r + gf1:r + gf1:pu
[[21]] accepted ~ 1 + r + gf1 + gf1:r + gf1:pu
[[22]] accepted ~ 1 + r + pu + gf1:r + gf1:pu
[[23]] accepted ~ 1 + pu + gf1 + pu:r
[[24]] accepted ~ 1 + r + pu + pu:r
[[25]] accepted ~ 1 + pu + gf1
[[26]] accepted ~ 1 + r + pu
[[27]] accepted ~ 1 + pu + gf1 + pu:r + gf1:r
[[28]] accepted ~ 1 + r + pu:r + gf1:r + gf1:pu
[[29]] accepted ~ 1 + r + gf1:r + gf1:pu
[[30]] accepted ~ 1 + pu + gf1 + pu:r + gf1:pu
[[31]] accepted ~ 1 + r + pu + pu:r + gf1:pu
[[32]] accepted ~ 1 + pu + gf1 + gf1:r
[[33]] accepted ~ 1 + gf1 + pu:r
[[34]] accepted ~ 1 + r + pu:r
[[35]] accepted ~ 1 + pu + pu:r + gf1:r
[[36]] accepted ~ 1 + r
[[37]] accepted ~ 1 + gf1
[[38]] accepted ~ 1 + pu + gf1 + pu:r + gf1:r + gf1:pu
[[39]] accepted ~ 1 + r + pu + gf1:pu
[[40]] accepted ~ 1 + pu + gf1 + gf1:pu
[[41]] accepted ~ 1 + gf1 + pu:r + gf1:r
[[42]] accepted ~ 1 + pu + gf1:r
[[43]] accepted ~ 1 + gf1 + gf1:r
[[44]] accepted ~ 1 + pu + pu:r + gf1:r + gf1:pu
[[45]] accepted ~ 1 + pu + gf1 + gf1:r + gf1:pu
[[46]] accepted ~ 1 + gf1 + pu:r + gf1:pu
[[47]] accepted ~ 1 + pu:r + gf1:r
[[48]] accepted ~ 1 + r + pu:r + gf1:pu
[[49]] accepted ~ 1 + r + gf1:pu
[[50]] accepted ~ 1 + gf1 + gf1:pu
[[51]] accepted ~ 1 + gf1:r
[[52]] accepted ~ 1 + pu + gf1:r + gf1:pu
[[53]] accepted ~ 1 + gf1 + pu:r + gf1:r + gf1:pu
[[54]] accepted ~ 1 + gf1 + gf1:r + gf1:pu
[[55]] accepted ~ 1 + pu + pu:r
[[56]] accepted ~ 1 + pu
[[57]] accepted ~ 1 + pu:r + gf1:r + gf1:pu
[[58]] accepted ~ 1 + gf1:r + gf1:pu
[[59]] accepted ~ 1 + pu + pu:r + gf1:pu
[[60]] accepted ~ 1 + pu:r
[[61]] accepted ~ 1
[[62]] accepted ~ 1 + pu + gf1:pu
[[63]] accepted ~ 1 + pu:r + gf1:pu
[[64]] accepted ~ 1 + gf1:pu
